# POCP-nf: an automatic Nextflow pipeline for calculating the percentage of conserved proteins in bacterial taxonomy

**DOI:** 10.1093/bioinformatics/btae175

**Published:** 2024-04-01

**Authors:** Martin Hölzer

**Affiliations:** Genome Competence Center (MF1), Robert Koch Institute, 13353 Berlin, Germany

## Abstract

**Summary:**

Sequence technology advancements have led to an exponential increase in bacterial genomes, necessitating robust taxonomic classification methods. The Percentage Of Conserved Proteins (POCP), proposed initially by Qin et al. (2014), is a valuable metric for assessing prokaryote genus boundaries. Here, I introduce a computational pipeline for automated POCP calculation, aiming to enhance reproducibility and ease of use in taxonomic studies.

**Availability and implementation:**

The POCP-nf pipeline uses DIAMOND for faster protein alignments, achieving similar sensitivity to BLASTP. The pipeline is implemented in Nextflow with Conda and Docker support and is freely available on GitHub under https://github.com/hoelzer/pocp. The open-source code can be easily adapted for various prokaryotic genome and protein datasets. Detailed documentation and usage instructions are provided in the repository.

## 1 Introduction

Advances in sequencing technologies have driven the genomics era and led to an unprecedented influx of bacterial genomes. Taxonomic classification of these genomes is crucial for understanding microbial diversity and evolutionary relationships. Various metrics are employed to delineate the taxonomy of bacteria, each providing unique insights into their genomic characteristics and evolutionary history. Typical metrics include Average Nucleotide Identity, digital DNA-DNA hybridization, and 16S rRNA gene sequence similarity (Hayashi Sant’Anna *et al.* 2019). These metrics offer valuable information but may exhibit limitations, especially in prokaryotes with high genomic plasticity. As such, researchers need to employ a combination of metrics to comprehensively assess the evolutionary relationships between bacterial taxa.

One such metric is the Percentage of Conserved Proteins (POCP), a genome-based measure of taxonomic diversity originally proposed by Qin *et al.* (2014). POCP quantifies the degree of protein conservation between two genomes, providing a measure of genomic similarity. Unlike metrics based solely on nucleotide sequences, POCP focuses on functional elements, offering a more biologically relevant perspective on genomic relatedness. Thus, POCP is particularly well-suited for assessing genus boundaries, a challenging task in prokaryotic taxonomy. By considering the conservation of proteins, which are critical players in cellular function, POCP offers a nuanced understanding of the genomic distinctions between bacterial genera. The metric complements other methods and contributes to a more comprehensive characterization of microbial taxonomy. In the past, POCP calculations have been used in combination with other metrics in various studies to assess the genus boundaries of prokaryotes (Pannekoek *et al.* 2016, Harris *et al.* 2017, Leclercq *et al.* 2019, Ormeño-Orrillo and Martínez-Romero 2019, Suresh *et al.* 2019, Esquivel-Elizondo *et al.* 2020, Lalucat *et al.* 2020, Miyake *et al.* 2020, Xu *et al.* 2020, Joshi *et al.* 2021, Meng *et al.* 2021, Pan *et al.* 2021, Vorimore *et al.* 2021, Azpiazu-Muniozguren *et al.* 2022), in metagenomic contexts (Lagkouvardos *et al.* 2016, Zou *et al.* 2019, Wylensek *et al.* 2020, Amulyasai *et al.* 2022), and even fungi (Wibberg *et al.* 2021). In all of these studies, the POCP calculations were implemented and carried out slightly differently, mainly using the criteria defined in the original publication by Qin *et al.* (2014).

To harmonize calculations for the assessment of genus boundaries and to make the results more reproducible and comparable, I present a Nextflow pipeline for the automatic calculation of POCP values called POCP-nf. The pipeline’s modular design allows seamless integration into larger analysis workflows, enabling researchers to leverage POCP alongside other metrics for a holistic exploration of bacterial evolutionary relationships. Through this contribution, I aim to enhance the accessibility and utility of POCP as a straightforward yet powerful metric in the rapidly evolving field of microbial genomics.

## 2 Pipeline description

The POCP-nf pipeline is developed in Nextflow (Di Tommaso *et al.* 2017), a workflow management system that ensures portability and scalability across different computing environments. The pipeline accepts bacterial genome or protein datasets in standard FASTA format as input, one per bacterial species. Protein-coding genes are predicted by Prokka (Seemann 2014). If protein sequences are provided, the protein annotation step is skipped.

The pipeline identifies orthologous proteins between species using the blastp subcommand and ‘ultra-sensitive’ mode of DIAMOND (Buchfink *et al.* 2015). Per default, the proteomes of two strains are compared by bidirectional all-vs-all orthology searches. The user can define an optional target genome or protein FASTA to switch to one-vs-all comparisons when needed and save runtime. Those proteins of the query genome that have a hit with an e-value of <1e−5, an identity of >40%, and an alignable region of >50% are called conserved based on the original POCP definition (Qin *et al.* 2014). Although the user can customize these parameters, I recommend sticking to the original parameters as defined by Qin *et al.* (2014) and otherwise clearly indicating any changed parameter options along with the version of POCP-nf used when sharing POCP results. The pipeline displays a warning if nonstandard parameters are used.

Each POCP value corresponds to the sum of the conserved proteins of two genomes divided by the sum of the total number of proteins of both genomes. A POCP of 50% was originally proposed as the genus limit, but it should be noted that the difference in proteome size between two strains influences the POCP value (Hayashi Sant’Anna *et al.* 2019).

The final output is a tab-separated table with all calculated pairwise POCP values and summary statistics to assess the results further. The modular design of the pipeline allows customization for specific datasets and enables integration into larger analysis workflows.

**Calculating alignments, BLASTP versus DIAMOND:** Please note that in the original POCP publication Qin *et al.* (2014) used BLASTP (Altschul *et al.* 1997) for calculating the alignments. However, DIAMOND is not only faster, which is an advantage when calculating POCP values for larger input datasets, but also achieves the sensitivity of BLASTP (Buchfink *et al.* 2021), especially when using the ‘ultra-sensitive’ mode, which is activated by default in POCP-nf. Another study comparing different alignment programs found that DIAMOND offered the best compromise between speed, sensitivity, and quality when a sensitivity option other than the default setting was selected (Hernández-Salmerón and Moreno-Hagelsieb 2020). I compared BLASTP and DIAMOND in ultra-sensitive mode within POCP-nf (v2.3.1) on five bacterial datasets with 15 to 167 genomes. I found an average difference in the percentage values of the calculated POCP of ∼0.16%. The runtime (protein input) for 44 *Enterococcus* genomes is halved from 10 h 12 m (POCP-nf with BLASTP) to 5 h 30 m (POCP-nf with DIAMOND) on a laptop with eight cores. Further details can be found in the GitHub manual. I have, therefore, decided to use DIAMOND as a more modern solution for calculating the alignments in POCP-nf.

## 3 Installation and usage

Only Nextflow and either Conda, Mamba, Docker, or Singularity for dependency handling are needed to run the POCP-nf pipeline. The pipeline can be installed and executed with just two commands:

# install pipeline

nextflow pull hoelzer/pocp

# run selected release on input genomes

nextflow run hoelzer/pocp -r 2.3.1\

--genomes ’<path/to/genomes/*fasta>’\

-profile local,docker

The repository’s documentation provides detailed instructions, more advanced commands, and dependencies. Customization options and parameters are documented to accommodate different input formats and analysis environments.

## 4 Example analysis

**Figure 1.**
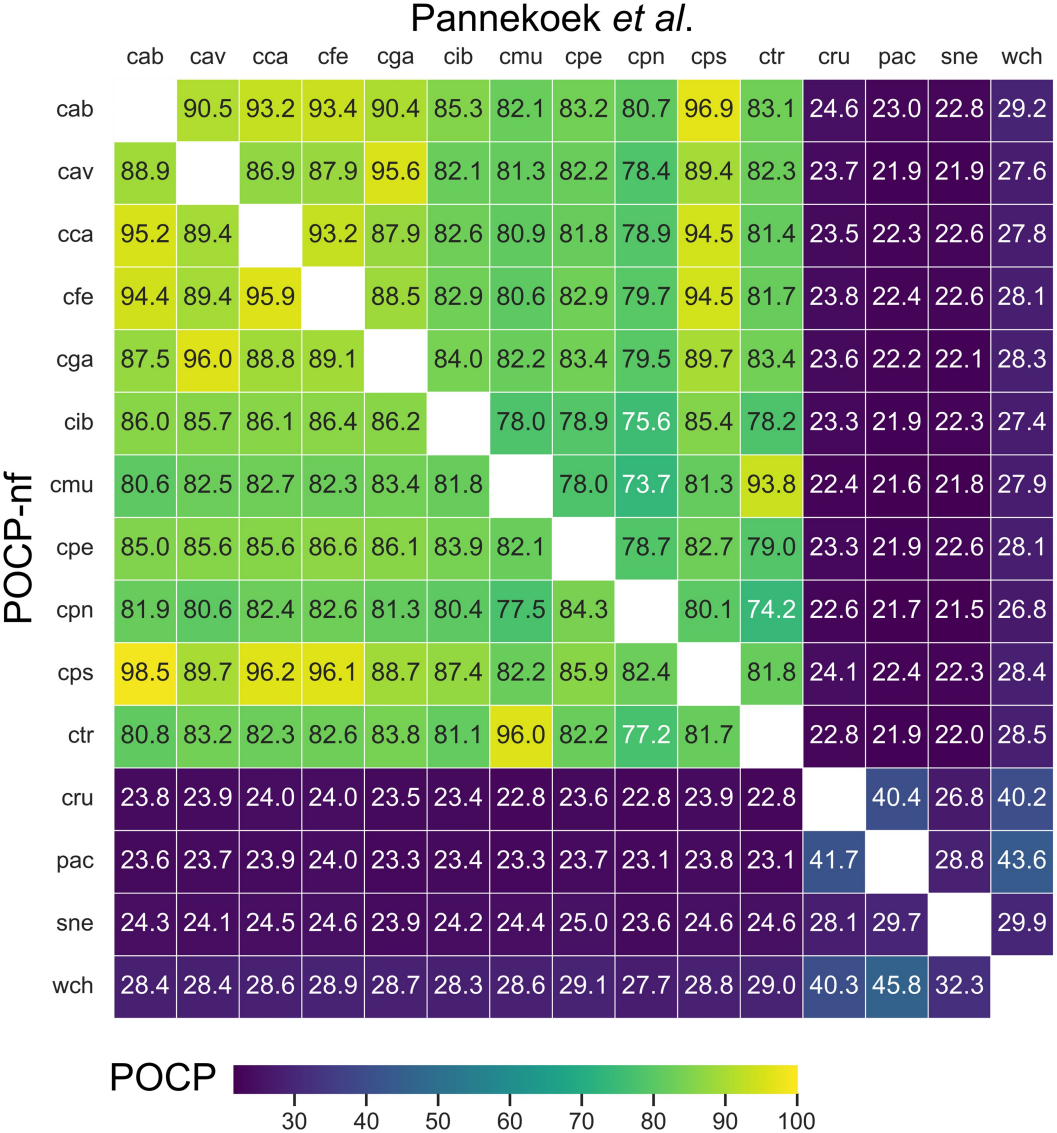
Pairwise POCP values from the original study of Pannekoek *et al.* (2016) (upper triangle) and recalculated with POCP-nf (lower triangle) of *Chlamydia* strains and outgroups. The average difference of percentage is 1.72% between all POCP values. *Chlamydia* (*C*.) *abortus* (cab), *C.avium* (cav), *C.caviae* (cca), *C.felis* (cfe), *C.gallinacea* (cga), *C.ibidis* (cib), *C.muridarum* (cmu), *C.pecorum* (cpe), *C.pneumoniae* (cpn), *C.psittaci* (cps), *C.trachomatis* (ctr), *Parachlamydia acanthamoebae* (pac), *Simkania negevensis* (sne), *Waddlia chondrophila* (wch), *Candidatus Rubidus massiliensis* (cru)

To showcase the pipeline performance and output, I re-analyzed genomic data of 15 species from a study about the genus delineation of *Chlamydiales* species, where the authors used POCP values to justify the reunifying of the genera *Chlamydia* and *Chlamydophila* into one single genus *Chlamydia* (Pannekoek *et al.* 2016). I obtained the genome FASTAs from NCBI based on Supplementary Table S1 from the previously mentioned study. The pipeline in version 2.3.1 ran 26 min on a Linux laptop with eight cores, using <2 GB RAM. Figure 1 shows the calculated POCP values from the study in 2016 (upper triangle) compared to the re-calculated POCP values using the Nextflow pipeline (bottom triangle). The POCP values differ slightly, most likely due to differences in the protein annotation used in 2016 and with POCP-nf, and underlines the importance of a uniform method for calculating comparable POCP values. Note that the same results can only be achieved if the same protein FASTAs are used as input for the same method (same tools, tool versions, and parameters). However, the resulting POCP values correspond to those calculated and published in 2016.

## 5 Conclusion

POCP can serve as a robust genomic index for defining genus boundaries for prokaryotic groups. However, it is also important to emphasize that POCP is only one genomic metric among others. Researchers must interpret the results in the context of additional analyses for a holistic understanding of prokaryotic taxonomy. For example, POCP with a standard cutoff of 50% was not suitable for delimiting taxa of the family *Bacillaceae* at the genus level (Aliyu *et al.* 2016) and cannot yield a single criterion for dividing the genus *Borrelia* into two genera (Gupta 2019).

In this context, I also want to mention Protologger (Hitch *et al.* 2021), an all-in-one genome description tool designed to simplify the data collection required to generate protologues. The software, available for local installation and as a Galaxy (Afgan *et al.* 2022) tool, can calculate various metrics, including POCP values. However, while Protologger is a comprehensive software package for various computations comprising taxonomic placement, functional annotations, and ecological analyses, applying it only for POCP calculations on larger datasets, integrating it with other pipelines, or running it on a high-performance cluster or in the cloud can be challenging. In addition, Protologger is associated with a high computing effort, a long installation routine, and high memory requirements if the user is only interested in POCP values. Another alternative for calculating POCP values is provided in the web service EDGAR3.0 (Dieckmann *et al.* 2021), a comparative genomics and phylogenomics platform hosted via Galaxy. EDGAR3.0 is also an easy-to-use web service, especially for nonexperts, but it is subject to restrictions like those mentioned above. For users unfamiliar with the command line interface, I recommend using web services such as Protologger and EDGAR3.0 for the POCP calculation. However, I would encourage them to use POCP-nf on the command line as the necessary installations are already reduced to a minimum by using Nextflow (see example above and GitHub manual).

The POCP-nf pipeline fills a crucial gap by providing a user-friendly, lightweight, locally installable, and automated tool for calculating and harmonizing the percentages of conserved proteins. By facilitating efficient taxonomic classification, researchers can leverage the pipeline to gain insights into genus boundaries based on genomic data.

## Data Availability

The pipeline is freely available at https://github.com/hoelzer/pocp. Input data and additional results for the example analysis are available at https://osf.io/2tzd9. A comparison between BLASTP and DIAMOND for POCP calculation is available in the GitHub manual.
